# Target product profile: *Trypanosoma brucei gambiense* test for low-prevalence settings

**DOI:** 10.2471/BLT.23.290176

**Published:** 2023-06-26

**Authors:** Gerardo Priotto, Jose R Franco, Veerle Lejon, Philippe Büscher, Enock Matovu, Joseph Ndung'u, Sylvain Biéler, Dieudonné Mumba, Nick Van Reet, Paul Verlé, Vincent Jamonneau, Pere P Simarro, Augustin Kadima Ebeja, Dieudonné Sankara, Daniel Argaw Dagne

**Affiliations:** a Control of Neglected Tropical Diseases, World Health Organization, Avenue Appia 20, 1211 Geneva 27, Switzerland.; b Institut de Recherche pour le Développement, CIRAD, University of Montpellier, France.; c Institute of Tropical Medicine, Antwerp, Belgium.; d College of Veterinary Medicine, Animal Resources and Biosecurity, Makerere University, Kampala, Uganda.; e Foundation for Innovative New Diagnostics-Kenya, Nairobi, Kenya.; f Neglected Tropical Diseases Programme, Foundation for Innovative New Diagnostics, Geneva, Switzerland.; g Department of Parasitology, Institut National de Recherche Biomédicale, Kinshasa, Democratic Republic of the Congo.; h World Health Organization Regional Office for Africa, Communicable Disease Unit, Brazzaville, Congo.

## Abstract

Having caused devastating epidemics during the 20th century, the incidence of life-threatening human African trypanosomiasis has fallen to historically low levels as a result of sustained and coordinated efforts over the past 20 years. Humans are the main reservoir of one of the two pathogenic trypanosome subspecies, *Trypanosoma brucei gambiense*, found in western and central Africa. The expected advent of a safe and easy-to-use treatment to be given to seropositive but microscopically unconfirmed individuals would lead to further depletion; in the meantime, the presence of *T. b. gambiense* infection in the community must be monitored to allow the control strategy to be adapted and the elimination status to be assessed. The World Health Organization has therefore developed a target product profile that describes the optimal and minimal characteristics of an individual laboratory-based test to assess *T. b. gambiense* infection in low-prevalence settings. Development of the target product profile involved the formation of a Neglected Tropical Diseases Diagnostics Technical Advisory Group and a subgroup on human African trypanosomiasis diagnostic innovation needs, and an analysis of the available products and development pipeline. According to the product profile, the test should ideally: (i) require a minimally invasive or non-invasive specimen, collectable at peripheral facilities by minimally trained health workers; (ii) demonstrate good sensitivity and high specificity; (iii) have a stability of samples allowing transfer to reference laboratories preferably without cold chain; (iv) be stable over a wide range of environmental conditions for more than 2 years; and (v) after marketing, be available at low cost for at least 7 years.

## Résumé

Après avoir causé des épidémies dévastatrices au cours du 20^e^ siècle, la trypanosomiase humaine africaine, potentiellement mortelle, a vu son incidence chuter à un niveau historiquement bas grâce aux efforts conjoints et soutenus déployés ces deux dernières décennies. Les humains constituent le principal réservoir de l'une des deux sous-espèces pathogéniques de trypanosome, *Trypanosoma brucei gambiense*, que l'on retrouve en Afrique occidentale et centrale. L'arrivée d'un traitement sûr et simple d'utilisation, qui serait administré aux individus séropositifs mais sans confirmation microscopique, devrait entraîner une nouvelle diminution; dans l'intervalle, la présence d'une infection à *T. b. gambiense* au sein de la communauté doit être surveillée afin de pouvoir adapter la stratégie de lutte et évaluer le statut d'élimination. Par conséquent, l'Organisation mondiale de la Santé a élaboré un profil de produit cible qui détaille les caractéristiques minimales et optimales d'un test individuel en laboratoire visant à confirmer l’infection à *T. b. gambiense* dans les régions à faible prévalence. La mise au point de ce profil a entraîné la formation d'un Groupe consultatif technique sur le diagnostic des maladies tropicales négligées et d'un sous-groupe consacré aux besoins en matière d'innovation diagnostique pour la trypanosomiase humaine africaine, qui a conduit une analyse des produits existants et des projets de développement. Selon le profil de produit, le test devrait idéalement: (i) nécessiter un prélèvement d'échantillon peu ou non invasif, pouvant être effectué dans des structures périphériques par des professionnels de la santé ayant reçu une formation sommaire; (ii) faire preuve d'un bon niveau de sensibilité et d’un niveau élevé de spécificité; (iii) avoir une stabilité des échantillons permettant le transfert vers des laboratoires de référence, de préférence sans chaîne de froid; (iv) rester stable dans un large éventail de conditions environnementales pendant plus de deux ans; et enfin, (v) après commercialisation, être disponible à bas coût pendant au moins sept ans.

## Resumen

Tras haber causado epidemias devastadoras durante el siglo XX, la incidencia de la tripanosomiasis humana africana potencialmente mortal ha descendido a niveles históricamente bajos gracias a los esfuerzos sostenidos y coordinados de los últimos 20 años. El ser humano es el principal reservorio de una de las dos subespecies patógenas del tripanosoma, *Trypanosoma brucei gambiense*, presente en África Occidental y Central. La prevista disponibilidad de un tratamiento seguro y fácil de administrar a personas seropositivas, pero no confirmadas al microscopio, permitiría una mayor eliminación; mientras tanto, se debe vigilar la presencia de la infección por *T. b. gambiense* en la comunidad para poder adaptar la estrategia de control y evaluar el estado de eliminación. Por consiguiente, la Organización Mundial de la Salud ha elaborado un perfil de producto objetivo que describe las características óptimas y mínimas de una prueba de laboratorio individual para evaluar la infección por *T. b. gambiense* en regiones de baja prevalencia. El desarrollo del perfil de producto objetivo implicó la formación de un Grupo de Asesoramiento Técnico sobre Diagnóstico de Enfermedades Tropicales Desatendidas y un subgrupo sobre las necesidades de innovación en el diagnóstico de la tripanosomiasis humana africana, así como un análisis de los productos disponibles y en desarrollo. Según el perfil objetivo, lo ideal sería que la prueba: (i) requiriera una muestra mínimamente invasiva o no invasiva, que pudiera ser recogida en centros periféricos por personal sanitario con una capacitación mínima; (ii) demostrara una buena sensibilidad y alta especificidad; (iii) tuviera una estabilidad de las muestras que permita su transferencia a laboratorios de referencia, preferiblemente sin cadena de frío; (iv) fuera estable en un amplio rango de condiciones ambientales durante más de 2 años; y (v) tras su comercialización, estuviera disponible a bajo coste durante al menos 7 años.

## ملخص

بعد أن تسبب داء النوم الأفريقي البشري المهدد للحياة في أوبئة مدمرة خلال القرن العشرين، انخفض معدل الإصابة به إلى مستويات منخفضة تاريخيًا نتيجة للجهود المستمرة والمنسقة على مدى العشرين عامًا الماضية. البشر هم العائل الرئيسي لواحد من الفصيلتين الفرعيتين المسببة لمرض داء النوم، داء النوم البروسي الغامبي، الموجود في غرب ووسط إفريقيا. إن الظهور المتوقع لعلاج آمن وسهل الاستخدام يتم إعطاؤه للأفراد الذين تكون نتيجة المصل لديهم إيجابية، ولكن من غير المؤكد مجهريًا إصابتهم، سيؤدي إلى مزيد من الاستهلاك؛ في أثناء ذلك، فإن وجود عدوى داء النوم البروسي الغامبي يجب مراقبتها في المجتمع للسماح لاستراتيجية المكافحة بالتكيف، وتقييم حالة القضاء على الداء. لذلك طورت منظمة الصحة العالمية ملف لتعريف المنتج المستهدف ليصف الخصائص المثلى والدُنيا لاختبار الفرد في المختبر لتقييم عدوى داء النوم البروسي الغامبي في المناطق ذات الانتشار المنخفض. تضمن تطوير ملف تعريف المنتج المستهدف تشكيل "مجموعة استشارية فنية لتشخيص أمراض المناطق المدارية المهملة"، ومجموعة فرعية معنية باحتياجات الابتكار التشخيصي لداء النوم الأفريقي البشري، وتحليل المنتجات المتاحة، وقنوات التطوير. وفقًا لملف تعريف المنتج، يجب أن يكون الاختبار من الناحية المثالية: (1) يتطلب عينة تستدعي تدخلاً جراحيًا طفيفًا، أو غير جراحية، يمكن جمعها في منشآت خارجية بواسطة عمال صحيين مدربين تدريباً محدوداً؛ و(2) يظهر حساسية جيدة وخصوصية عالية؛ و(3) تتمتع باستقرار في العينات يسمح بنقلها إلى مختبرات مرجعية ويفضل أن يكون ذلك بدون سلسلة تبريد؛ و(4) أن يكون مستقرًا في وجود مجموعة كبيرة من الظروف البيئية لمدة أكثر من عامين؛ و(5) يكون متاحًا بعد التسويق بتكلفة منخفضة لمدة 7 أعوام على الأقل.

## 摘要

致命的非洲人类锥虫病在 20 世纪造成了灾难性的传染病，但经过过去 20 年的持续共同努力，该病的发病率已降至历史最低水平。*布氏冈比亚锥虫*是在非洲西部和中部发现的两种致病性锥虫的亚种之一，人类是其主要宿主。针对血清反应阳性但未通过显微镜观察进行确认的个体，预期推出的治疗方法安全且易于使用，将进一步消除该病的传播；与此同时，必须监测社区中是否存在*布氏冈比亚锥虫*感染，以便调整防控策略并评估消除情况。因此，世界卫生组织制定了一份目标产品简介，描述了在低流行环境中评估*布式冈比亚锥虫*感染情况的单独实验室检测的最佳属性和最小属性。制定目标产品简介，涉及成立一个被忽视热带病诊断技术咨询小组和一个满足非洲人类锥虫病诊断创新需求的小组，并对现有产品和开发渠道进行分析。根据产品简介，理想的测试应具有以下属性：(I) 需要使用微创或非侵入式样本采集方法，可由受过最低限度培训的卫生工作者在外围移动设备中采集；(ii) 表现出较好的灵敏度和较高的特异性；(iii)有稳定的样本允许在没有冷链的情况下转移到参考实验室；(iv) 在广泛的条件下能保持稳定性超过 2 年；(v) 上市后，可维持低成本供应至少 7 年。

## Резюме

Являясь причиной ужасающих эпидемий в 20-м веке, заболеваемость опасным для жизни человека африканским трипаносомозом снизилась до исторически низкого уровня в результате последовательных и скоординированных усилий за последние 20 лет. Люди являются основным естественным резервуаром одного из двух патогенных подвидов трипаносом *Trypanosoma brucei gambiense*, встречающихся в Западной и Центральной Африке. Появление безопасного и простого лечения, которое можно будет назначать лицам с серопозитивным, но микроскопически неподтвержденным статусом, приведет к дальнейшему снижению заболеваемости, тем временем необходимо будет отследить наличие инфекции *T. b. gambiense* в соответствующей общине, чтобы можно было адаптировать стратегию борьбы и оценить статус элиминации. В связи с этим Всемирной организацией здравоохранения был разработан целевой профиль продукта, который описывает оптимальные и минимальные характеристики отдельных лабораторных испытаний для оценки инфекции *T. b. gambiense* в условиях низкой распространенности. Разработка целевого профиля продукта включала создание технической консультативной группы по диагностике забытых актуальных заболеваний и подгруппы по инновационным потребностям диагностики африканского трипаносомоза человека, а также анализ имеющихся продуктов и стратегии развития. В соответствии с профилем продукта тест при идеальных условиях должен: (i) требовать использования образцов с минимальным инвазивным или неинвазивным воздействием, собираемых в условиях периферийных учреждений медицинскими работниками с минимальной подготовкой; (ii) демонстрировать хорошую чувствительность и высокую специфичность; (iii) обеспечивать стабильность образцов, позволяющую транспортировать их до эталонной лаборатории, предпочтительно без использования холодовой цепи; (iv) быть стабильным в широком диапазоне условий окружающей среды в течение более 2 лет; (v) после выхода на рынок быть доступным по низкой цене в течение как минимум 7 лет.

## Introduction

Human African trypanosomiasis, endemic in sub-Saharan Africa, is a life-threatening parasitic infection transmitted by the tsetse fly.^1^ Having caused devastating epidemics during the 20th century, its incidence has fallen to historically low levels as a result of sustained and coordinated efforts over the past 20 years.^2^ The disease is caused by two trypanosome subspecies with distinct epidemiology. (i) *Trypanosoma brucei rhodesiense*, found in eastern and southern Africa, is harboured by wild and domestic animals (which constitute its reservoir) and is occasionally transmitted to humans; and (ii) *T. b. gambiense*, which is found in western and central Africa, has its main reservoir in humans and between 2011 and 2020 accounted for 95% (32 275 out of 34 096 reported cases) of the total caseload of human African trypanosomiasis.^2^

Because clinical signs and symptoms of human African trypanosomiasis are unspecific, diagnosis relies on laboratory techniques.^3^ Field serodiagnostic tests exist only for *T. b. gambiense* and are based on the detection of antibodies; they are therefore not confirmatory of infection. With the current low disease prevalence, the positive predictive value of serological tests is particularly low. Field-applicable tools include the card agglutination test for trypanosomiasis,^4^ used mainly in active screening by specialized mobile teams, and the rapid diagnostic tests that are more suitable for individual testing at point-of-care. Confirmation of *T. b. gambiense* infection requires microscopic examination of body fluids, necessitating specific training. The best performing methods are laborious and demonstrate 85%−95% diagnostic sensitivity when performed by skilled personnel.^5^

With regards to the control and elimination of gambiense human African trypanosomiasis, it has long been observed that a strategy of repeated rounds of screening followed by treatment of detected cases can bring down the prevalence substantially.^6^ The expected advent of a safe and easy-to-use treatment to be given to seropositive but microscopically unconfirmed individuals would lead to further depletion of the parasite reservoir.^7^ However, if this type of treatment policy is applied, the presence of *T. b. gambiense* infection in the community must be monitored to allow the control strategy to be adapted in each setting and to provide key data to assess the elimination status in endemic countries.

The World Health Organization (WHO) has therefore developed a target product profile that describes the optimal and minimal characteristics of an individual laboratory-based test to assess *T. b. gambiense* infection in low-prevalence settings. The purpose of this profile is to inform product developers of the key performance specifications and characteristics of such a test that will meet the needs of end-users in sub-Saharan African countries. The main features of such a product include: (i) its use at an individual level in suspects (e.g. those with serological or clinical suspicion, and/or geographical proximity to confirmed cases) to determine if they are currently infected (ideally) or have previously been infected by *T. b. gambiense*; (ii) high specificity and good sensitivity; (iii) simple specimen collection by minimally trained staff, and stability of samples allowing transfer to reference laboratories preferably without cold chain; and (iv) ideally requiring blood from finger-prick with minimal sample processing. To help with interpretation, it should also be established for how long the test may remain positive in an individual after a *T. b. gambiense* infection has cleared (e.g. antibody tests may remain positive for years).

## Methods

The development of this target product profile was led by the WHO Department of Control of Neglected Tropical Diseases following standard WHO guidance. To identify and prioritize diagnostic needs, a WHO Neglected Tropical Diseases Diagnostics Technical Advisory Group was formed. Subgroups to advise on specific neglected tropical diseases were subsequently created, including a subgroup working on human African trypanosomiasis diagnostic innovation needs. This group of independent experts comprised leading international scientists and specialists, including from countries where the disease is endemic, who followed standard WHO declaration of interest procedures. 

A landscape analysis of the available products and development pipeline was conducted, and salient areas with unmet needs were identified. Through meetings and remote consultations, use-cases were developed for the hypothetical tools considered as the four main gaps, and assigned an order of priority. A template adapted to the human African trypanosomiasis context was agreed, and used to develop the target product profile. The draft of this target product profile (rated as third-highest priority) underwent several rounds of review by the subgroup members, before being reviewed by the members of the advisory group. A draft version was posted on the WHO website for public consultation on 23 September 2022 for 28 days, inviting viewers to complete a proforma comment form. After further review by members of the advisory group, a final version of the target product profile was made available online.^8^

## Target product profile

### Intended use

This test is intended for individuals at increased suspicion for gambiense human African trypanosomiasis after serological testing or clinical examination, or because of proximity to a confirmed case. Ideally, a positive test should indicate current infection with gambiense human African trypanosomiasis, with inclusion of a previous infection as acceptable. 

It would be desirable for a test to detect the presence of *T. b. gambiense*-specific antibodies, *T. b. gambiense* antigens, or whole-parasite or *T. b. gambiense*-specific nucleic acids; however, the detection of antibodies or antigens of *Trypanozoon*, or whole-parasite or *Trypanozoon*-specific nucleic acids, would be minimally acceptable. The sampling should be conducted before treatment in the case of molecular or antigen analytes. Antibodies may persist from a previous infection, which would allow the retrospective diagnosis of *T. b. gambiense* infection; sampling could be conducted immediately after treatment in this case.

The type of specimen required should ideally be either minimally invasive (e.g. finger-prick or venous blood) or non-invasive (e.g. saliva, urine or tears). A test feasible at a national or sub-national laboratory would be ideal, but the need to refer to international laboratories would be minimally acceptable. A trained health worker (with ideally < 1 hour’s basic training, or acceptably up to 4 hours) should be capable of collecting the specimens. At the reference laboratory performing the test, a 1–2 days’ training for laboratory personnel should be sufficient, and a maximum of 7 days would be acceptable.

### Assay performance

A clinical sensitivity of at least 95% is desired (> 90% minimally acceptable), comparable to the most sensitive parasitological tests currently in use. False negatives resulting in non-treatment not only have a risk of death but also result in lower prevalence estimates. Further, because false positives result in unnecessary treatment and overestimated prevalence, a clinical specificity of at least 99% is desired (> 95% minimally acceptable). 

Because diagnosis and treatment are currently based on microscopy at the subgenus level, a *Trypanozoon*-specific test would be acceptable; however, the test should ideally detect lower taxa (e.g. *T. b. gambiense* type 1). In terms of detection thresholds, an analytical sensitivity of 10 parasites/mL or less is desired (although ≤ 50 parasites/mL would be minimally acceptable). Repeatability and reproducibility should be as high as possible, with *Κ*-values of greater than 0.96 and 0.94 desired, respectively (> 0.92 and > 0.90 minimally acceptable, respectively). Control of functionality is required, and the availability of temperature-stable positive and negative controls for batch and kit testing is desired.

### Operational characteristics

The test should ideally be usable in conditions of 10 °C–40 °C and 10%–88% relative humidity, although margins of 10°C –30 °C and 40%–70% would be minimally acceptable. Preparation of the specimen in the field should preferably be a single-step process (maximum four steps), with preferably no (or minimal) need for precision liquid handling or specialized material. Specimens should ideally be testable individually (i.e. without any waste of unused reagent), although testing in batches of less than eight would be minimally acceptable. Results should ideally be available within 48 hours (maximum 1 week) after arrival at the test laboratory, preferably with automatic scoring, recording and integration of results (visual/manual scoring, recording and integration minimally acceptable). 

### Reagent and sample handling

The stability of the reagent should consider the time frame for distribution from manufacturer, passage through customs and the limited number of tests that may need to be performed in low-prevalence settings. Tests should be stable at 4 °C–45 °C (4 °C–8 °C minimally acceptable) and 40%–88% relative humidity, ideally for more than 2 years (1 year minimally acceptable). Ideally, individual tests (or < 8-specimen series) should be accompanied by all necessary accessories and reagents for processing. Reagents should either be ready for use, or else usable after a preferred maximum of two steps (maximum five steps minimally acceptable).

Specimen processing (volume ≤ 0.07 mL for finger-prick; ≤ 5 mL for venous blood) before preferably non-urgent transport (e.g. 4 weeks) should ideally be achievable either without any further steps or a maximum of two steps. Stability of the sample for 4 weeks at 40 °C or 12 months at 4 °C is desirable; stability of the sample for 3 days at 35 °C or 1–2 months at 4 °C would be minimally acceptable. Sample waste management procedures should adhere to standard biosafety measures for handling potentially infectious materials, with waste disposal in biosafety bins and sharps containers following standard guidelines.

### Commercial aspects

The supply of the test should be guaranteed for preferably 7 years or more (at least 5 years minimally acceptable) after marketing, and manufacturers should replace non-functioning tests or instruments. External support should be available with a desired response time of ideally 1 day (1 week maximum).

Individual tests should be available to health facilities at a maximum cost of 5 United States dollars (US$); the minimally acceptable cost is under US$ 20.

## Conclusion

Currently, the diagnostic tools available in or near the field are not appropriate for determining *T. b. gambiense* infection because of the low sensitivity of simple microscopy tests, and the low feasibility of more sophisticated tests with higher performance. According to the product profile, the test should ideally: (i) require a minimally invasive or non-invasive specimen, collectable by fixed or mobile facilities close to affected communities by minimally trained health workers; (ii) demonstrate good sensitivity, high specificity, repeatability and reproducibility; (iii) have a simple specimen collection procedure, and a good stability of specimens allowing transfer to reference laboratories preferably without cold chain ; (iv) be stable over a wide range of temperature and relative humidity for more than 2 years; and (v) after marketing, be available at low cost for at least 7 years. In settings with an absence of performant microscopy, treatment could be decided based on the results of this test. This test would also become very useful in the framework of a potential strategy of providing immediate treatment to unconfirmed serological suspects, a situation that is currently predicted with the potential advent of an oral, single-dose safe treatment. In such situations, this test could confirm or rule out *T. b. gambiense* infection a posteriori, allowing the epidemiological situation to be monitored.
